# Evaluating the Effectiveness of Interventions to Improve the Follow-up Rate for Children With Visual Disabilities in an Eye Hospital in Nepal: Nonrandomized Study

**DOI:** 10.2196/43814

**Published:** 2023-02-23

**Authors:** Manisha Shrestha, Gopal Bhandari, Sureshkumar Kamalakannan, Gudlavalleti Venkata Satyanarayana Murthy, Suresh Kumar Rathi, Anirudh Gaurang Gudlavalleti, Varun Agiwal, Hira Pant, Binod Pandey, Ramesh Ghimire, Daman Ale, Sajani Kayastha, Rakshya Karki, Daya Shankar Chaudhary, Raghunandan Byanju, Operational Research Capacity Building Study Group

**Affiliations:** 1 Bharatpur Eye Hospital Bharatpur Metropolitan City, Chitwan Nepal; 2 Indian Institute of Public Health Hyderabad India; 3 see Acknowledgments

**Keywords:** counseling, follow-up, intervention study, Nepal, ophthalmology, pediatrics, public health

## Abstract

**Background:**

Monitoring ocular morbidity among pediatric patients requires regular follow-up visits. We found that the follow-up rate was poor among children in our setting. Therefore, we intended to assess the effectiveness of 2 interventions—(1) counseling and (2) SMS text messaging and phone calls—to improve the follow-up rates.

**Objective:**

This study aimed to evaluate the effectiveness of 2 interventions, counseling and SMS and phone calls group, as well as a routine standard care for improving the follow-up rate of pediatric patients.

**Methods:**

A Nonrandomized, quasiexperimental design was used. Children (aged 0-16 years) with ocular conditions requiring at least 3 follow-up visits during the study period were included. A total of 264 participants were equally allocated to the 3 intervention groups of (1) counseling, (2) SMS and phone calls, and (3) routine standard care group. A 20-minute counseling session by a trained counselor with the provision of disease-specific leaflets were given to those in the counseling group. For the second intervention group, parents of children received an SMS text 3 days before and a phone call 1 day before their scheduled follow-up visits. Participants allocated for the routine standard care group were provided with the existing services with no additional counseling and reminders. Participants attending 3 follow-ups within 2 days of the scheduled visit date were considered compliant. The difference in and among the proportion of participants completing all 3 follow-up visits in each group was assessed.

**Results:**

The demographic characteristics of the participants were similar across the study groups. Only 3% (8/264) of participants completed all 3 follow-up visits, but overall compliance with the follow-up, as defined by the investigators, was found to be only 0.76% (2/264). There was no statistically significant difference in the proportion of follow-up between the intervention groups. However, the proportion of participants attending the first and second follow-ups, as well as the overall total number of follow-ups, was more in the SMS and phone-call group followed by the counseling group.

**Conclusions:**

We did not find any evidence on the effectiveness of our interventions to improve the follow-up rate. The primary reason could be that this study was conducted during the COVID-19 pandemic. It could also be possible that the intensity of the interventions may have influenced the outcomes. A rigorously designed study during the absence of any lockdown restrictions is warranted to evaluate intervention effectiveness. The study also provides useful insights and highlights the importance of designing and systematically developing interventions for improving the follow-up rate and ensuring a continuum of care to children with visual disabilities in Nepal and similar contexts.

**Trial Registration:**

ClinicalTrials.gov NCT04837534; https://clinicaltrials.gov/ct2/show/NCT04837534

**International Registered Report Identifier (IRRID):**

RR2-10.2196/31578

## Introduction

### Background

Childhood visual impairment and blindness remain an important public health issue. An estimated 1.4 million children globally are blind [1]. The Nepal Pediatric Ocular Disease study in 2014 estimated a childhood blindness and severe visual impairment (visual acuity<6/60) prevalence of 70/100,000 [2]. Another study in the Narayani Zone of Nepal in 2017 estimated the prevalence of childhood blindness and severe visual impairment as 30/100,000 and moderate visual impairment as 25/100,000 [3]. Increasing the global knowledge base and planning for effective childhood eye care services is a top priority to enable children with visual impairment to realize their full visual potential [4]. Follow-up of pediatric patients is important for their regular ocular morbidity monitoring, especially for amblyopia management [4,5].

The pediatric eye care teams at Bharatpur Eye Hospital (BEH) observed that there was poor adherence to follow-up visits of children with visual impairment. An exploratory analysis of data during the first week (January 1, 2019, to January 7, 2019) revealed that follow-up compliance was very low among children aged 0 to 16 years in the pediatric department. Among the children advised for follow-up, only 22% were found to have come for at least one follow-up visit. A problem analysis showed that a lack of awareness in children and their parents regarding the importance of follow-up and patients forgetting the dates of the follow-up visit (usually when there is a long gap for follow-up) may be the major contributing factors to poor adherence to follow-up.

A study from India revealed distance and cost as major barriers, as was the inability of the eye care center to communicate the importance of follow-up [6]. Another study conducted in Nepal found poor follow-up rates for patients following pediatric cataract surgery, which, however, improved after the implementation of a high-quality pediatric counseling service, a follow-up program, a tracking system, and phone reminders [7]. Many studies have compared different methods of reminder options such as telephone calls, email, and SMS text messages to improve compliance with follow-up [8-12]. A study done to improve the neonatal follow-up showed that the monthly first-visit show rate increased from 60% to 76% during the intervention period, and 75% of families who received parent education presented for their initial visit, compared to 51% of families who did not receive parent education [13]. Although several interventions were experimented in previous studies, it is still not clear which kinds and components of the interventions were influencing treatment effectiveness. It is also documented that the interventions and the definition of follow-up differed in terms of intensity, duration, and time, respectively. Therefore, in order to improve the follow-up of children with visual impairment in BEH and Nepal in general, we intended to assess the effectiveness of different kinds of interventions to improve follow-up in these client groups.

### Primary Objective

The aim of this paper is to assess the effectiveness of 2 different interventions, namely counseling and SMS and phone calls, against routine standard care to improve follow-up of children with visual impairment in BEH Nepal.

## Methods

### Study Design

This is a nonrandomized, quasiexperimental study.

### Setting

This study was conducted at the Hiralal Santudevi Pradhan Institute of Ophthalmic Sciences. BEH is a centrally located tertiary eye hospital in the Chitwan district of Nepal.

### Participants

The participants of this study were selected considering the following criteria:

- Children with a visual impairment, aged 0-16 years
- Enrolled in the pediatric department of BEH
- Diagnosed with ocular conditions requiring at least 3 follow-up visits
- Supported by parents or guardians having a mobile phone who can use the mobile phone and read SMS texts

### Intervention

#### Counseling Group

The parent or guardian and the child received a 20-minute counseling session from a trained counselor (SK) as per a structured counseling protocol at every follow-up visit where the disease-specific leaflet was used as a counseling tool, a copy of which was also handed over to them. The counseling protocol for common ocular conditions had been designed by the research team. Children, along with their parents or guardians, received counseling irrespective of participant age, parental education, ocular conditions, and other factors. If more than one guardian or both parents accompanied the child, both were included in the counseling session. The counselor delivered verbal counseling for all participants in all follow-up visits irrespective of the ocular conditions and other factors.

#### SMS Text Message and Phone Call Group

The parents of children received an SMS text 3 days before and a phone call 1 day before their scheduled follow-up visits. Text messages were sent until it was confirmed that the message had been received. In the case of message delivery failure, it was sent 3 more times to ensure successful delivery. A phone call was deemed to be completed once it was received by the respondent; calls were repeated at least 3 times if the phone was not answered in the first or second instance. If the call was not answered even after 3 attempts, the participant was excluded from the study.

#### Routine Standard Care Group or Control Group

In this group, the children underwent visual acuity testing and refraction by an optometrist. The pediatric ophthalmologist performed a detailed ocular examination and advised necessary investigations to diagnose and formulate a treatment plan. Basic counseling was done by the consultant regarding the ocular condition, treatment, and need for follow-up. No additional counseling or reminders were offered to these patients.

### Compliance to Follow-up

The participants’ first visit to the hospital and the 3 scheduled follow-up visits needed to be completed for them to be considered compliant with the follow-up. Only those participants who completed the first follow-up were considered for the second follow-up, and only those who came for the second follow-up were considered for the third follow-up. This definition was adopted from a protocol used in an earlier study in Nepal [7].

The patients were considered compliant to follow-up only if they came within the window period of (+/–) 2 days close to their scheduled visit. The rescheduling of the next follow-up date was calculated from the attended date as per the follow-up schedule for each ocular condition. The purpose of observing compliance to follow-up was to determine the impact of counseling and reminders through SMS texts and phone calls on the increment of the proportion of children completing their 3 follow-up visits based on the developed proforma and to find out if the differences in the proportion in the follow-up rate between the 3 different groups were statistically significant.

### Data Analysis

Data were processed and analyzed using Excel (Microsoft Corp) and STATA version 14.2. (Stata Corp) The results were presented in terms of frequency counts with percentages for categorical variables. The significant association or difference between different intervention groups was measured by chi-square with degree of freedom for qualitative variables. A *P* value of less than .05 was considered significant.

### Ethical Considerations

Ethics approval was obtained from the Ethical Review Board (ERB) of the Nepal Health Research Council (ERB protocol registration #761/2020 P and ClinicalTrials.gov No: NCT04837534). Written consent was taken from the children in an assent form (if aged 9 years or older) and from their parents or guardians in a consent form before enrolling them in the study. All the information collected was secured and stored safely by the chief investigators. The data were completely anonymized for the purpose of privacy and confidentiality. The participants were not compensated for participating in the study.

## Results

### Demographic Characteristics

A total of 264 participants were enrolled in this study. The participants were divided randomly (alternate sequence) and were equally allocated into 3 study groups. Routine standard care group, SMS text message and phone call group, and the counseling group. Demographic characteristics of the participants, such as gender, age groups, ethnicity, parental educational status, parents or guardian accompanying the child, and occupation of parents were equally distributed among the study groups, and the difference was not statistically significant. About 62% (n=164) of the participants were male, and 38% (100) were female. Factors such as the total distance from the hospital, the time taken, and the cost incurred for the travel by participants to reach the hospital in the 3 study groups were also not statistically significant. The baseline characteristics of the participants in each group were not very different. Table 1 provides the details of the participants’ demographic characteristics.

**Table 1:** Sociodemographic profile of the participants based on different study groups (N=264).

Characteristics		Routine standard care, n (%)	Counseling, n (%)	SMS and phone call, n (%)	Total, n (%)	Chi-square (*df*)		*P* value	
**Gender**							0.2254 (2)		.89
	Male	56 (63.64)	53 (60.23)	55 (62.50)	164 (62.12)				
	Female	32 (36.36)	35 (39.77)	33 (37.50)	100 (37.88)				
**Age group**							4.36 (2)		.11
	≤8 years	58 (65.91)	69 (78.41)	68 (72.27)	195 (73.86)				
	>8 years	30 (34.09)	19 (21.59)	20 (22.73)	69 (26.14)				
**Parents or guardian**							1.9003 (4)		.75
	Mother	58 (21.96)	58 (21.96)	67 (25.37)	183 (69.31)				
	Father	17 (6.43)	19 (7.19)	14 (5.30)	50 (18.93)				
	Others	13 (4.92)	10 (3.78)	7 (2.65)	30 (11.36)				
**Ethnicity**							0.7858 (2)		.68
	Aryan	54 (61.36)	54 (61.36)	49 (55.68)	157 (59.47)				
	Mongol and others	34 (38.64)	34 (38.64)	39 (44.34)	107 (40.53)				
**Educational status of parent or guardian**							8.87 (6)		.18
	Illiterate	10 (11.36)	6 (6.82)	10 (11.36)	26 (9.85)				
	Primary	5 (5.68)	11 (12.50)	7 (7.95)	23 (8.71)				
	Secondary and higher secondary	62 (70.45)	54 (61.36)	49 (55.68)	165 (62.50)				
	Bachelor and above	11 (12.50)	17 (19.32)	22 (25)	50 (18.94)				
**Occupation**							0.9126 (2)		.63
	Non–income-generating occupation	53 (6023)	59 (67.05)	55 (62.50)	167 (63.26)				
	Income-generating occupation	35 (39.77)	29 (32.95)	33 (37.50)	97 (36.74)				
**Distance (km)**							2.39 (4)		.66
	0-50	76 (86.36)	76 (86.36)	71 (80.68)	223 (84.47)				
	51-100	6 (6.82)	6 (6.82)	6 (6.82)	18 (6.82)				
	>100	6 (6.82)	6 (6.82)	11 (12.86)	23 (8.71)				
**Time taken (min)**							5.2414 (4)		.26
	0-30	59 (67.05)	60 (68.18)	56 (63.64)	175 (66.29)				
	31-60	17 (19.32)	19 (21.59)	13 (14.77)	49 (18.56)				
	>60	12 (13.64)	9 (10.23)	19 (21.59)	40 (15.15)				
**Cost of 2-way travel (NR^a^)**							4.002 (4)		.41
	0-100	40 (45.45)	49 (55.68)	43 (48.86)	132 (50.00)				
	101-500	40 (45.45)	30 (34.09)	32 (36.36)	102 (38.64)				
	>500	8 (9.09)	9 (10.23)	13 (14.77)	30 (11.36)				

^a^NR: Nepalese Rupee (US $1=NR 132).

### Impact of the Intervention on Follow-up

This study did not find any statistically significant difference between the study groups during the follow-ups (first follow-up chi-square: *χ*^2^_2_=3.19, *P*=.20; second follow-up chi-square: *χ*^2^_2_=0.92, *P*=.62; and third follow-up chi-square: *χ*^2^_2_=0.25, *P*=.86). However, the proportion of participants attending the first and second follow-ups as well as the overall total number of follow-ups was more in the SMS and phone call group followed by the counseling group. In the routine standard care group, only 3 participants attended all 3 follow-up visits, and out of them, only 1 participant attended all the follow-ups on schedule. Similarly, 3 participants in the counseling group and 2 participants in the SMS and phone call attended all 3 follow-ups, respectively. Among them, none of the participants attended the follow-up on schedule in the counseling group, and only 1 participant was on schedule in the SMS and phone call group. The overall compliance with the follow-up as defined by the investigators was found to be 0.76% (2/264). Table 2 provides more details related to the participants’ follow-up. Except for the first follow-up, it was also observed that most participants who attended the second and third follow-ups attended it after the schedule.

The cost for 2-way travel between the groups was also not statistically significant (Table 3). A comprehensive cost-effectiveness analysis of the interventions is being carried out, and its results will be published as a separate paper.

Comparing the compliance to the follow-up with some specific demographic characteristics showed no statistically significant difference (Table 4). About 50%-55% of parents or guardians who had secondary or higher secondary education attended the first, second, and third follow-ups. Male children were brought to the follow-ups more in all 3 groups compared to female children. Among the participants who attended all 3 follow-ups, all were accompanied by their mothers. About 65% (127/195) of the participants who attended the follow-ups belonged to the non–income-generating group.

**Table 2:** Impact of the interventions on follow-up.

Variables			Routine standard care (n=88), n (%)		Counselling (n=88), n (%)		SMS and phone call (n=88), n (%)		Total (N=264)		Chi-square (*df*)			*P* value	
**F/u^a^ category**															
	Advised for f/u	88 (100)		88 (100)		88 (100)		N/A^b^		N/A					
	First f/u attended	24 (28.92)		25 (28.41)		34 (38.64)		83 (31.44)		3.19 (2)			.20		
	Second f/u attended	9 (10.23)		10 (11.36)		13 (14.77)		32 (12.12)		0.92 (2)			.62		
	Third f/u attended	3 (3.41)		3 (3.41)		2 (2.27)		8 (100)		0.25 (2)			.86		
	Total f/u visits	36 (29.27)		38 (30.89)		49 (39.84)		123 (100)		2.83 (2)			.24		
**Actual attended date at first f/u (n=83)**												5.57 (4)			.23
	Prior to schedule	6 (25.00)		5 (20.00)		3 (8.82)		14 (16.87)							
	On schedule	10 (41.67)		9 (36.00)		21 (61.76)		40 (48.19)							
	After schedule	8 (33.33)		11 (44.00)		10 (29.41)		29 (34.94)							
**Actual attended date at second f/u (n=32)**												3.25 (4)			.51
	Prior to schedule	2 (22.22)		0		2 (15.38)		4 (12.50)							
	On schedule	1 (11.11)		3 (30.00)		4 (30.77)		8 (25.00)							
	After schedule	6 (66.67)		7 (70.00)		7 (53.85)		20 (62.50)							
**Actual attended date at third f/u (n=8)**												4.00 (4)			.40
	Prior to schedule	0		1 (33.33)		1 (50.00)		2 (25.00)							
	On schedule	1 (33.33)		0		1 (50.00)		2 (25.00)							
	After schedule	2 (66.67)		2 (67.67)		0		4 (50.00)							

^a^F/u: follow-up.

^b^N/A: not applicable.

**Table 3:** Travel cost with follow-up attendance (N=264).

Variables			Presenting, n (%)		First f/u^a^, n (%)		Chi-square (*df*)			*P* value			Second f/u, n (%)			Chi-square (*df*)			*P* value			Third f/u, n (%)			Chi-square (*df*)			*P* value	
**Cost category (NP^b^)**								4.84 (2)			.09						5.73 (2)			.06						5.15 (2)			.08
	Up to 100	132 (50.00)		36 (43.37)								13 (40.63)									1 (12.50)								
	101-500	102 (38.64)		40 (48.19)								18 (56.25)									6 (75.00)								
	>500	30 (11.36)		7 (8.43)								1 (3.13)									1 (3.03)								

^a^F/u: follow-up.

^b^NP: Nepalese Rupees; cost of 2-way travel per person (US $1=NR 132).

**Table 4:** Comparison of compliance to follow-up rate with sociodemographic profile.

Characteristics		First f/u^a^ attended (n=83), n (%)	*P* value		Second f/u attended (n=32), n (%)		*P* value		Third f/u attended (n=8), n (%)		*P* value	
**Gender**				.34				.26				.45
	Male	55 (66.27)			17 (53.13)				6 (75)			
	Female	28 (33.73)			15 (46.88)				2 (25)			
**Age group**				.16				.79				.94
	≤8 years	66 (79.52)			23 (71.88)				6 (75)			
	>8 years	17 (20.48)			9 (28.13)				2 (25)			
**Parents or guardian**				.14				.90				.16
	Mother or both parents	62 (79.49)			23 (79.31)				8 (100)			
	Father	16 (20.51)			6 (20.69)				0			
	Others	5 (1.89)			3 (1.13)				0			
**Ethnicity**				.15				.69				.58
	Aryan	44 (53.01)			18 (56.25)				4 (50)			
	Mongol and others	39 (46.99)			14 (43.75)				4 (50)			
**Educational status of parent or guardian**				.31				.21				.90
	Illiterate	10 (12.05)			3 (9.38)				1 (12.50)			
	Primary	9 (10.85)			1 (3.13)				1 (12.50)			
	Secondary and higher secondary	45 (54.22)			18 (56.25)				4 (50)			
	Bachelor and above	19 (22.89)			10 (31.25)				2 (25)			
**Occupation**				.68				.21				.48
	Non–income-generating group	51 (61.45)			17 (53.13)				6 (75)			
	Income-generating group	32 (38.55			15 (46.88)				2 (25)			

^a^F/u: follow-up.

## Discussion

### Principal Findings

This study aimed to assess the effect of counseling as one intervention and SMS and phone call together as another intervention compared to the routine standard care (control group) to increase the follow-up rate of children with visual impairment in 1 tertiary care hospital in Nepal, and it did not find any statistically significant difference between the study groups on the follow-up rate. The baseline data on follow-up in the pediatric department from January 2019 was 22%, which we assumed would be improved to 50% with our intervention; however, compliance to follow-up, as defined in our study, was very low (ie, 0.76%). Nevertheless, compared to the standard as well as counseling group, the proportion of participants in the first, second, and third follow-up combined was more in the SMS and phone call group. This was only applicable if the strict definition for compliance to follow-up was not applied.

There may be several reasons for poor compliance to follow-up in this study. One of the possible reasons could be the COVID-19 pandemic and the lockdown restriction imposed during the time when participant recruitment took place. Fear of potential risks of COVID-19 infection as well as the serious health and socioeconomic consequences of breaching the pandemic restrictions might have influenced compliance to follow-up.

The other potential reason could be the intensity and the timing of the intervention. The content of the counseling intervention and the duration of 20 minutes might not have been effective enough to produce a sizeable effect on the outcomes of this study. This is very similar to the number of calls, the content of the conversation, and the acceptability of the text messages by the participants. Given that the intervention was developed based on previous experience rather than systematic development as recommended by the Medical Research Council guidelines, the factors that could influence treatment effectiveness may have been missed out. Lastly, the affordability of the participants for 3 continued follow-ups as recommended by the investigator team may also have been a factor to consider. This is of immense interest to the investigators, and a comprehensive study is in progress to understand the effect of costs on compliance with follow-up.

Different studies have shown varied results regarding the effect of these kinds of interventions on people with visual impairments. Follow-up rates have been found to improve with these types of interventions in some studies, while other studies show no significant improvement [7-21]. For example, a similar study conducted in Nepal to improve follow-up among pediatric patients with cataract found that the rate of follow-up for first, second, and third follow-up visits increased from 87% to 96%, 60% to 81%, and 37% to 57% without and with the intervention, respectively [7]. However, there was a full-time pediatric counselor, a tracking system, and a cell phone reminder used as intervention packages, which are different from the interventions used in this study.

This study has several implications. Firstly, there is a need to systematically develop interventions to address the growing needs of people with visual impairments, particularly in the community. Given the economic situation of most participants in a country such as Nepal, continuum of care through sustainable interventions must be explored further. Given the complexity of the intervention, it is also essential to have a dedicated team trained exclusively to focus on follow-up and community-based care rather than using the task transfer or multitasking approach to address the needs of persons with visual disabilities. This study also highlights the need for an inclusive program during the pandemic that must be organized by the government for people with visual impairment in a country such as Nepal, considering the risks and consequences of the pandemic on this vulnerable group.

### Limitations

Similar to other studies on this topic, there are certain limitations. The study was quasiexperimental in design, and a controlled clinical trial would be a rigorous design to evaluate the effects accurately. Recruitment during the pandemic, especially for an outcome related to follow-up, may not seem feasible. However, this study has provided the opportunity to understand the disadvantages of recruitment for a study related to follow-up during lockdown travel restrictions. This knowledge will help the investigators conduct a feasibility study before embarking on a sufficiently powered, large clinical trial. Lastly, a very strict definition of compliance to follow-up could have also been a reason for poor compliance.

Visual impairment is an important public health problem in Nepal. Given the geographical and attitudinal barriers to accessing specific evidence-based eye care services, it is important to sensitize people experiencing disability due to visual impairments about the importance of the continuum of care. Similarly, it is also very important to build the capacity of institution-based teams to develop pathways, protocols, and effective interventions to address the unmet needs of people with visual impairments in Nepal through inclusive and affordable strategies.

## Abbreviations

BEH: Bharatpur Eye Hospital

## Data Availability

The data collected have been presented in the manuscript. Any request for additional data will be reviewed by the advisory committee and will be shared upon request.
